# The molecular mechanisms of gas signaling molecules in bronchopulmonary dysplasia related lung injury

**DOI:** 10.3389/fcell.2026.1861860

**Published:** 2026-07-21

**Authors:** Lulu Zhang, Lanbo Hu, Fangrui Ding

**Affiliations:** 1 Department of Neonatology, Tianjin Central Hospital of Gynecology Obstetrics, Tianjin, China; 2 Tianjin Key Laboratory of Human Development and Reproductive Regulation, Tianjin, China; 3 Department of Neonatology, Nankai University Maternity Hospital, Tianjin, China

**Keywords:** bronchopulmonary dysplasia, endogenous small-molecule, gas signaling molecules, lung injury, mechanism

## Abstract

Bronchopulmonary dysplasia (BPD) is the most prevalent chronic respiratory disease in preterm infants. Its pathogenesis is linked to multiple factors including lung immaturity secondary to preterm birth, hyperoxia exposure, mechanical ventilation-induced injury, and inflammatory responses. Characterized by alveolar simplification and abnormal pulmonary vascular development as the core pathological features, BPD exerts a profound negative impact on the long-term prognosis of affected infants. As endogenous small-molecule substances, gas signaling molecules play a pivotal role in regulating BPD related lung injury by virtue of their extensive biological properties. Among these molecules, nitric oxide (NO), carbon monoxide (CO), and hydrogen sulfide (H_2_S) have been subjected to extensive and in-depth investigations. This narrative review summarizes the mechanisms through which the above-mentioned gas signaling molecules modulate BPD-related lung injury. More importantly, this review comprehensively discusses the influences of crosstalk among the three gas signaling molecules on BPD. It objectively elaborates the protective effects of gas signaling molecules in BPD-related lung injury as well as the limitations of their clinical application. The findings lay a theoretical foundation for developing refined and individualized clinical prevention and treatment regimens of BPD based on gas signaling molecules in future clinical practice.

## 1 Introduction

Bronchopulmonary dysplasia (BPD) is the most common and severe respiratory complication in preterm infants, especially in extremely preterm infants. The globally reported incidence of BPD ranges from 10% to 89% (10%–73% in Europe, 18%–89% in North America, 18%–82% in Asia, and 30%–62% in Oceania), which seriously affects the survival rate and long-term quality of life of affected infants (Gilfillan et al., 2021; Siffel et al., 2021; Enzer et al., 2024). With the advancement of neonatal intensive care, the mortality rate of preterm infants has decreased significantly. However, the incidence of BPD has not declined. On the contrary, it has even shown an upward trend. Severe BPD is not only associated with high mortality but also increases the risk of long-term complications involving the respiratory system, nervous system, and growth and development (Jeon et al., 2025; Jenkinson and Dassios, 2026). The prevention and treatment of BPD remain a great challenge at present, mainly due to its complex pathogenesis that involves multiple processes, including immature lung development, oxidative stress, inflammatory response, and abnormal tissue repair (Ambalavanan et al., 2026).

**FIGURE 1:**
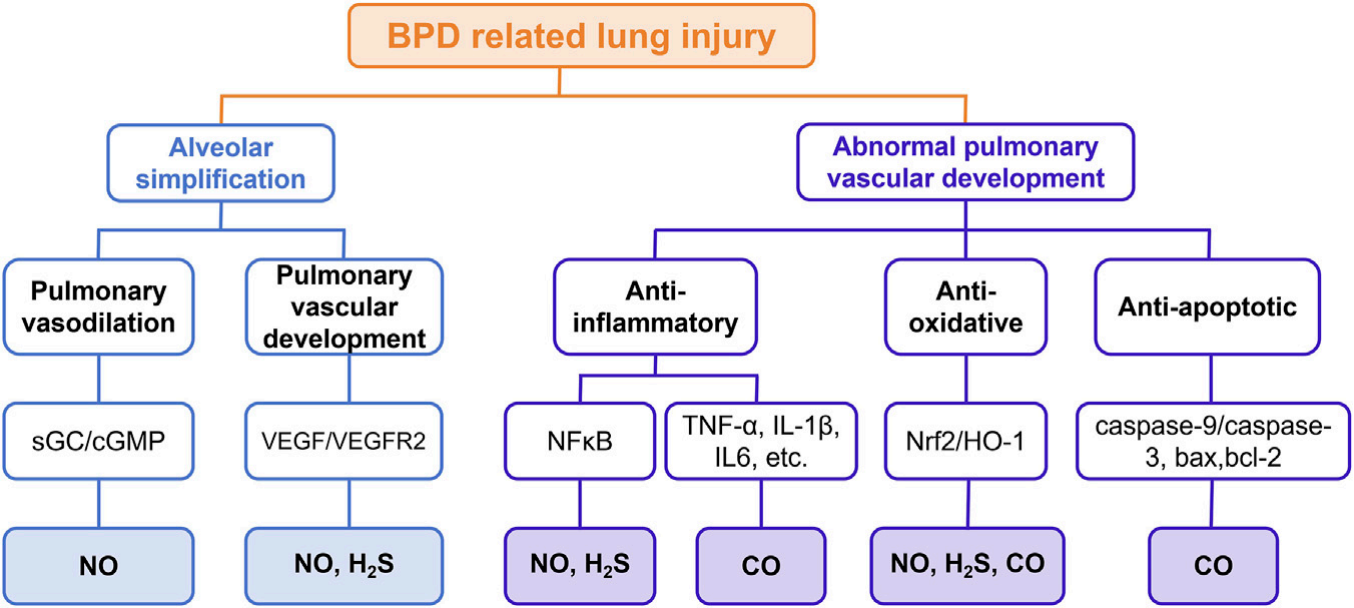
The molecular mechanisms of gas signaling molecules in BPD related lung injury.

Gas signaling molecules are a class of endogenously enzymatically generated small gaseous molecules, characterized by high lipid solubility, free permeability across cell membranes, independence of specific membrane receptors, specific target sites, and the ability to exert unique physiological functions at physiological concentrations (Jiang et al., 2024). Currently, the gas signaling molecules studied in relation to BPD mainly include nitric oxide (NO) (Zheng et al., 2023; Feng et al., 2024; Fraga et al., 2023), carbon monoxide (CO) (Anyanwu et al., 2014; Lin and Wang, 2020), and hydrogen sulfide (H_2_S) (Ganguly et al., 2021; Madurga et al., 2014; Madurga et al., 2015), which play critical roles in physiological regulation and pathological injury repair in the body. Recent studies have demonstrated that gas signaling molecules exert important protective effects in BPD-related lung injury by regulating signaling pathways related to oxidative stress, inflammatory response, angiogenesis, and alveolar development. This article reviews the molecular mechanisms of the three major gas signaling molecules in BPD-related lung injury (Figure1). By elaborating their potential mechanisms as well as their clinical applications and limitations, this article clarifies their non-negligible theoretical value in the prevention and treatment of BPD-related lung injury. This as well carries great significance for the long-term research and clinical translation of gas signaling molecules.

## 2 Characteristics of BPD-related lung injury and properties of gas signaling molecules

### 2.1 Clinical background and pathological characteristics of BPD related lung injury

Over the past half century, advances in perinatal medicine, along with the adoption of gentler ventilation strategies, the administration of antenatal glucocorticoids and postnatal exogenous pulmonary surfactant, have led to substantial changes in the phenotype of BPD. In terms of the pathological basis of the disease, in contrast to the “classic BPD' described by Northway et al., which is characterized by structural destruction of alveoli and respiratory tract as well as pulmonary fibrosis, the typical features of the current “new BPD' are mainly manifested as alveolar simplification, abnormal development of pulmonary microvessels, and altered thickness of alveolar septa (Cassady et al., 2020; Course et al., 2026; Gilfillan and Bhandari, 2023). These pathological changes are accompanied by impaired pulmonary mechanical function (decreased lung compliance and increased airway resistance) and reduced gas exchange efficiency, and may lead to long-term sequelae such as pulmonary arterial hypertension and chronic respiratory diseases in the later stage (Moschino et al., 2021; Caskey et al., 2016).

### 2.2 Etiological factors of BPD-related lung injury

The etiological factors of BPD-related lung injury mainly include antenatal high-risk factors, postnatal direct injury factors, and other secondary factors (Taglauer et al., 2018; Bonadies et al., 2020). Antenatal high-risk factors increase the susceptibility of postnatal lung injury primarily by impairing lung tissue development, among which preterm birth and low birth weight are the core prerequisites for BPD occurrence. Extremely preterm infants with a gestational age of less than 28 weeks and a birth weight of less than 1,000 g have lung tissue that is still in the canalicular or saccular stage. Their alveolar epithelial cells (especially type II alveolar epithelial cells) are immature in differentiation, with insufficient synthesis of pulmonary surfactant. Meanwhile, the pulmonary microvascular network is not fully formed, and the vascular endothelial barrier function is fragile, making it highly vulnerable to damage from external stimuli (Bush et al., 2023). Maternal complications during pregnancy, such as gestational hypertension, pre-eclampsia, and gestational diabetes mellitus, can lead to insufficient placental perfusion, placing the fetus in a state of chronic hypoxia and nutritional deficiency (Diniz et al., 2023). This condition inhibits the expression of key lung development factors including vascular endothelial growth factor (VEGF) and fibroblast growth factor (FGF). Maternal infection and inflammation, such as intra-amniotic infection, are important risk factors for BPD (Yu et al., 2024; Hundscheid et al., 2023). Maternal infection can activate fetal inflammatory pathways through the placenta, leading to elevated levels of pro-inflammatory cytokines (TNF-α, IL-1β, IL-6) in fetal lung tissue, disrupting the alveolar epithelial-endothelial barrier and interfering with the process of alveolarization (Gras-Le Guen et al., 2008; Kunzmann et al., 2007; Menshykova and Dobryanskyy, 2025). Rational antenatal administration of glucocorticoids can promote fetal lung maturation; however, repeated or excessive use can inhibit the normal proliferation and differentiation of lung tissue, resulting in a reduction in the number of alveoli and an increased risk of pulmonary interstitial fibrosis (Grier and Halliday, 2004; Zhang et al., 2022). Postnatal factors are the direct drivers of BPD lung injury, which damage the structure of the immature lungs of preterm infants mainly through the triple mechanisms of mechanical injury, oxidative stress, and inflammatory response. Mechanical ventilation is an important means of respiratory support for preterm infants, but inappropriate ventilation strategies can cause severe ventilator-associated lung injury (Liu et al., 2025). Excessively high peak inspiratory pressure (PIP) and tidal volume can lead to overdistension and rupture of alveoli, triggering interstitial edema and inflammatory infiltration, and causing barotrauma or volutrauma. An inappropriately low positive end-expiratory pressure (PEEP) setting results in repeated alveolar collapse and re-expansion, damaging alveolar epithelial and vascular endothelial cells and inducing atelectrauma (Akyildiz et al., 2025; Dumpa et al., 2025; Deguise and Lemyre, 2025). Mechanical stimulation activates inflammatory signaling pathways in lung tissue, releasing a large number of pro-inflammatory cytokines that exacerbate the cascade reaction of lung injury and cause biotrauma (Smalley et al., 2025). The antioxidant enzyme system (superoxide dismutase, glutathione peroxidase, et al.) in the lung tissue of preterm infants has low activity. High-concentration oxygen therapy generates excessive reactive oxygen species, which attack the lipids, proteins, and DNA of alveolar epithelial and vascular endothelial cells, leading to cell apoptosis and pulmonary interstitial fibrosis. Even short-term exposure to high oxygen levels may result in long-term pulmonary vascular remodeling and stagnation of alveolar development (Yang et al., 2021; Lee et al., 2025).

Postnatal infection is the key driver of BPD progression. Nosocomial infections such as ventilator-associated pneumonia and sepsis involve direct invasion of lung tissue by pathogens (bacteria, viruses), which activate the innate immune response and release pro-inflammatory cytokines, exacerbating lung injury. Aseptic inflammation, such as damage-associated molecular patterns released after lung tissue injury, can continuously activate inflammatory pathways, forming a vicious cycle of “injury—inflammation—re-injury' (Salimi et al., 2022; Shima et al., 2022). Other secondary factors include abnormal pulmonary vascular development and genetic susceptibility.

### 2.3 Research significance of gas signaling molecules

Gas signaling molecules refer to a class of endogenous small-molecule substances that exist in gaseous form, can diffuse freely in organisms, and mediate intercellular or intracellular signal transduction (Table1). Their core characteristics include no requirement for carrier transport, ability to penetrate biological membranes, and broad range of action targets. At present, three gas signaling molecules have been extensively studied in the context of BPD-related lung injury, namely, nitric oxide (NO), carbon monoxide (CO), and hydrogen sulfide (H_2_S) (Lin and Wang, 2020; Davis and Pluth, 2025). Endogenous gas signaling molecules possess extensive physiological regulatory functions, and exert protective effects in BPD-related lung injury by modulating processes such as oxidative stress, inflammatory response, angiogenesis, and alveolar development. NO is generated by the catalysis of L-arginine by nitric oxide synthase (NOS). NOS is divided into three subtypes: neuronal NOS (nNOS), inducible NOS (iNOS), and endothelial NOS (eNOS). Its core functions involve regulating vasodilation, inhibiting platelet aggregation, participating in neural signal transmission, and modulating immune-inflammatory responses. In the respiratory system, NO can regulate airway tone and participate in pulmonary vascular remodeling (Nowaczyk et al., 2021; Gonzalez et al., 2025). CO is mainly produced by the catalytic decomposition of heme by heme oxygenase (HO). HO is classified into inducible HO (HO-1) and constitutive HO (HO-2). HO-1 expression can be induced by oxidative stress, inflammatory factors, and other stimuli. Its core functions include anti-inflammation, anti-oxidation, anti-apoptosis, and regulation of vascular tone. In lung injury, CO can alleviate alveolar epithelial cell apoptosis and inhibit the release of inflammatory factors (Kashfi and Patel, 2022; Ryter, 2022). H_2_S is mainly produced via catalytic synthesis by three enzymes: cystathionine-β-synthase (CBS), cystathionine-γ-lyase (CSE), and 3-mercaptopyruvate sulfurtransferase (3-MST). Among these, CSE is widely distributed in the cardiovascular and respiratory systems. Its core functions consist of vasodilation, inhibition of smooth muscle proliferation, anti-inflammation, anti-oxidation, and regulation of airway mucus secretion. In lung injury models such as BPD, H_2_S can alleviate lung tissue fibrosis and inflammatory infiltration (Jiang et al., 2024; Ganguly et al., 2021; Song et al., 2023).

**TABLE 1:** Multidimensional comparison of the effects of NO, CO and H₂S on BPD-related lung injury.

Comparison dimension	Nitric oxide (NO)	Carbon monoxide (CO)	Hydrogen sulfide (H₂S)
Enzymes for generation	Physiological eNOS/nNOS; pathological iNOS.	Constitutive HO-2; inducible HO-1	Main synthetic enzyme CSE CBS and 3-MST.
Major pathways	Protection: Activates sGC/cGMP to relax blood vessels, mildly activates Nrf2 and inhibits NF-κB; damage: Generates ONOO⁻ with O₂⁻, causes nitrosative damage, over-activates NF-κB to amplify inflammation	Antioxidant: Activates nrf2/HO-1 positive feedback loop for potent antioxidant effect Anti-inflammatory: Blocks NF-κB p65 nuclear translocation, inhibits NLRP3 inflammasome; resist fibrosis: Weakly activates sGC/cGMP, downregulates TGF-β to resist fibrosis	Antioxidant: Activates Nrf2, promotes GSH synthesis, scavenges ROS/ONOO⁻; anti-inflammatory: downregulates pulmonary levels of NF-α, iIL-1α/β, IL-2 and MIP-1, inhibits NF-κB activation
Protective effects	Relaxes pulmonary blood vessels, reduces pulmonary artery pressure, improves PPHN in preterm infants; mildly inhibits inflammation, promotes microvascular regeneration, slightly improves alveolar development, shortens ventilator dependency	Antioxidant, reduces oxidative damage induced by hyperoxia; anti-inflammatory, regulates macrophage polarization to M2 phenotype; inhibits myofibroblast activation, reduces collagen deposition, resists fibrosis Protects pulmonary endothelial barrier, maintains. alveolar structural stability	Promoting alveolar development, reverses alveolar simplification induced by hyperoxia, increases the number of secondary septa; anti-inflammatory and antioxidant, antagonizes NO-mediated nitrosative damage; protects mitochondria, inhibits epithelial apoptosis; improves microcirculation, resists fibrosis, facilitates lung repair
Harmful effects	Excessively generates ONOO⁻, destroys the alveolar-capillary barrier, aggravates pulmonary edema; amplifies inflammation, inhibits epithelial proliferation, promotes fibrosis; clinical risks: methemoglobinemia, bleeding tendency, pulmonary hemorrhage, inhibits normal lung development	High concentration competitively binds to hemoglobin, increases COHb, causes systemic hypoxia, damages heart and brain tissues; inhibitsmitochondrial cytochrome c oxidase, blocks respiratory chain, causes ATP depletion and cell necrosis; extremely high concentration inhibits epithelial repair, synergizes with hyperoxia to Aggravate oxidative damage	Supraphysiological concentration inhibits mitochondrial respiratory chain, causes cellular energy failure and toxicity; high concentration reversely induces airway/pulmonary artery spasm, aggravates pulmonary circulation disorder; mildly activates inflammasome, aggravates late interstitial inflammation
Evidence type	Sufficient preclinical evidence, verified mechanism in multi-species BPD models and NOS knockout experiments; abundant but conflicting clinical evidence, only BPD infants with concurrent PPHN benefit, no unified positive conclusion for simple prevention	Highly consistent preclinical evidence, with clear protective effects in cell/animal BPD models; scarce clinical evidence, no large-scale RCT of BPD in neonates	Good preclinical evidence, verified protective effect in cell/animal BPD models, CSE knockout can induce pulmonary dysplasia; extremely limited clinical evidence, only confirmed that plasma H₂S level is significantly reduced in BPD infants, no interventional RCT.
Translational barriers	Extremely narrow dose window, difficult for precise titration in preterm infants; limited efficacy, only benefits the subgroup with pulmonary hypertension; safety risks such as methemoglobin, bleeding and neurotoxicity; extremely short half-life, single administration route, high heterogeneity and poor reproducibility of clinical trials	Prominent safety concerns, preterm infants’ heart and brain are extremely sensitive to hypoxia, difficult to define the safe threshold of COHb; no standardized administration regimen; scarce bedside real-time monitoring equipment; high operational risk of gaseous administration, no neonatal safety data for new donors	Immature administration system, gaseous H₂S has high toxicity and strong irritation, slow-release donors only stay in animal experiments; narrow safe dose window, no pharmacokinetic/toxicokinetic data in preterm infants; short half-life, easy to be inactivated, unable to maintain effective concentration in the lung; lack of local precise monitoring means, no evidence-based basis for clinical intervention in neonates

## 3 Core pathophysiological basis of BPD-related lung injury

The core pathophysiological changes of BPD-related lung injury can be summarized as alveolar developmental arrest, abnormal pulmonary vascular development, persistent inflammation-oxidative stress injury, and the vicious cycle formed by the interaction of these three processes. These changes ultimately lead to lung structural remodeling and permanent impairment of lung function.

### 3.1 Alveolar developmental arrest and simplification of lung structure

Following birth, normal preterm infants should enter the critical stage of alveolarization, which is characterized by rapid expansion of alveolar numbers, thinning of alveolar walls, and maturation of secondary pulmonary lobules. However, the core structural abnormality of BPD is the arrest of alveolarization, specifically manifested as reduced alveolar numbers, increased alveolar volume, failure of primitive alveolar sacs to differentiate into mature alveoli, and cystic changes in lung tissue, resulting in a significant decrease in gas exchange area (Cui and Fu, 2024). The direct cause of this change is the impaired function of type II alveolar epithelial cells (AECII): their capacity to differentiate into type I alveolar epithelial cells (AECI) is inhibited by inflammatory factors, leading to disruption of the integrity of the alveolar epithelial barrier and failure to support the maturation of alveolar structures (Wu et al., 2024). Alveolar septa become thickened. Under injury stimulation, lung fibroblasts are abnormally activated and transformed into myofibroblasts, which secrete large amounts of type I/III collagen and extracellular matrix (ECM). This results in fibrosis of alveolar walls and decreased elasticity. Among the regulatory pathways involved, the TGF-β/Smad pathway is the core regulatory pathway of this process. Inflammatory factors can continuously activate this pathway, exacerbating ECM deposition and reducing lung structural compliance (Fan et al., 2025; Cederqvist et al., 2005; Kunzmann et al., 2018).

### 3.2 Abnormal pulmonary vascular development: deficient angiogenesis and vascular remodeling

Pulmonary vascular development is highly coupled with alveolar development, and vascular endothelial growth factor (VEGF) secreted by vascular endothelial cells is a key factor regulating alveolarization (Lun et al., 2025; Wickramasinghe et al., 2025). The pathophysiological changes of pulmonary vessels in BPD mainly involve two aspects. On one hand, there is insufficient formation of pulmonary microvessels. Pulmonary vascular endothelial cells of preterm infants have weak proliferation and differentiation abilities, and the expression of the VEGF/vascular endothelial growth factor receptor 2 (VEGFR2) signaling pathway is downregulated. This leads to sparse pulmonary microvascular networks and reduced vascular branching, which fail to provide sufficient nutrition and oxygen supply for alveolar development, further aggravating alveolarization arrest (Hu et al., 2024; Oak and Hilgendorff, 2017). On the other hand, high airway pressure from mechanical ventilation and high-concentration oxygen therapy directly damage vascular endothelium and inhibit the activation of the VEGF pathway (Perrone et al., 2023). Inflammatory factors (e.g., IL-6) also block angiogenesis by downregulating VEGFR-2 expression (Kim et al., 2010). Pulmonary microvascular remodeling is associated with pulmonary hypertension. Chronic hypoxia and inflammatory stimulation induce abnormal proliferation of pulmonary arteriolar smooth muscle cells (SMC), thickening of vascular walls, and luminal stenosis, leading to obstructive vascular lesions that progress to BPD-related pulmonary hypertension (BPD-PH). The core mechanism of this process is the excessive activation of the hypoxia-inducible factor-1α (HIF-1α) pathway. Upregulation of HIF-1α under hypoxic conditions promotes the proliferation of vascular SMC. Meanwhile, the imbalance of the endothelin-1/endothelin receptor system exacerbates vasoconstriction and remodeling, further elevating pulmonary artery pressure (Zhu et al., 2024).

### 3.3 Persistent inflammation and oxidative stress injury: core drivers of the pathological process

Inflammation and oxidative stress injury are the key links initiating and maintaining BPD-related lung injury, as well as the core bridge connecting alveolar developmental arrest and vascular abnormalities. The corresponding pathophysiological changes are characterized by inflammatory responses and an imbalance in the oxidant-antioxidant system. Maternal intra-amniotic infection is the primary risk factor for BPD (Hundscheid et al., 2023; Yamamoto et al., 2024; Staude et al., 2023). Pathogenic microorganisms or toxins enter the fetal body through the placenta, activating innate immune cells such as pulmonary macrophages and neutrophils in the fetus, and inducing the release of pro-inflammatory factors including TNF-α, IL-1β, and IL-6, thereby prematurely triggering pulmonary inflammatory responses (Xu et al., 2025; Cai et al., 2025). After birth, factors such as mechanical ventilation, oxygen therapy, and ventilator-associated pneumonia further activate immune cells, resulting in a “second hit' effect. This leads to sustained release of inflammatory factors and chemokines, exacerbating lung tissue injury. Regarding the imbalance between oxidative stress and the antioxidant system: the antioxidant enzyme system (e.g., superoxide dismutase, glutathione peroxidase) in the lungs of neonates is immature. Meanwhile, high-concentration oxygen therapy and mechanical ventilation induce excessive production of reactive oxygen species (ROS). ROS not only directly damage the DNA, proteins, and lipids of alveolar epithelial and vascular endothelial cells, but also activate the nuclear factor-κB pathway, further promoting the release of pro-inflammatory factors and forming a vicious cycle of inflammation-oxidative stress (Guan et al., 2025; Wang et al., 2026; Teng et al., 2025).

## 4 Molecular mechanisms of major gas signaling molecules in BPD-related lung injury

### 4.1 Molecular mechanisms of NO in BPD-related lung injury

The mechanism underlying the effect of NO on BPD is dual-sided. On the one hand, it can alleviate lung injury via mechanisms such as vasodilation, anti-inflammation and antioxidation, on the other hand, excessive NO production may induce oxidative stress and amplify inflammation, thereby exacerbating lung tissue remodeling.

The protective effects and mechanisms of NO against BPD are mainly reflected in two aspects: first, promoting pulmonary vasodilation and pulmonary vascular development; second, inhibiting inflammatory responses and oxidative stress. The endothelial nitric oxide synthase catalyzes the production of endogenous NO, which activates soluble guanylate cyclase (sGC) in vascular smooth muscle cells to raise cyclic guanosine monophosphate (cGMP). This dilates pulmonary arterioles, lowers pulmonary artery pressure and boosts pulmonary perfusion (Gonzalez et al., 2025). A series of studies on pulmonary hypertension animal models, pulmonary hypertension with concurrent cardiopulmonary injury, and patients with heart failure that aimed to reduce pulmonary pressure all confirmed that activating the NO-sGC-cGMP signaling pathway can effectively dilate the pulmonary arteries and is the key mechanism for regulating pulmonary vascular tension (Nubbemeyer et al., 2025; Triposkiadis et al., 2022). In the related research on the BPD rat model, it was found that NO can promote the proliferation, differentiation and migration of pulmonary microvascular endothelial cells by upregulating the VEGF/VEGFR2 pathway, accelerate the formation of new pulmonary vascular networks. This, in turn, promotes the development of pulmonary vessels in BPD rats and improves BPD-related lung injuries (Lu et al., 2015). In addition, excessive pro-inflammatory factors (e.g., TNF-α, IL-1β) and ROS are the key factors leading to alveolar epithelial cell injury and alveolarization arrest in BPD (Xu et al., 2025; Cai et al., 2025). NO can inhibit the activation of the nuclear factor-κB pathway to reduce the release of pro-inflammatory factors, meanwhile, it alleviates oxidative stress-induced lung tissue damage by scavenging superoxide anions (Guan et al., 2025; Wang et al., 2026; Teng et al., 2025). Preclinical studies have found that exogenous supplementation of low-concentration NO can reduce the activation level of alveolar macrophages in preterm rats, decrease neutrophil infiltration, and alleviate lung tissue fibrosis (Laube et al., 2017).

The damaging effects and mechanisms of NO on BPD lie in the fact that when NO is overproduced or reacts with superoxide anions, it is converted into peroxynitrite anion (ONOO^−^), which induces oxidative damage and inflammatory amplification, thus exacerbating the pathological progression of BPD (Zhou et al., 2003). Inducible nitric oxide synthase (iNOS) is extensively activated in the inflammatory microenvironment of BPD, and the excessive NO produced reacts with ROS to generate ONOO^−^. This substance has strong oxidizing properties, which can damage the DNA, proteins and lipids of alveolar epithelial cells and vascular endothelial cells, leading to cell apoptosis and lung tissue remodeling. Studies on mouse models have found that excessive NO can inhibit the proliferation and differentiation of lung fibroblasts, reduce the synthesis and deposition of extracellular matrix (e.g., collagen, elastin), hinder the formation of secondary alveolar septation, and ultimately lead to alveolarization arrest (Xu et al., 2022).

In animal models, iNO improves gas exchange and pulmonary structural development, but the efficacy of NO in the clinical treatment of BPD remains controversial. Most clinical trials have demonstrated that inhaled NO fails to reduce the incidence, severity, or associated mortality of BPD, nor does it affect pulmonary function or oxygenation status during the progression of BPD, nor decrease the utilization rate of mechanical ventilation (Abiramalatha et al., 2022; Mirza et al., 2025; Hascoet et al., 2005). Small-sample randomized controlled trials have shown that early inhalation of low-concentration iNO (5–20 ppm) can reduce the incidence of BPD and improve long-term pulmonary function in preterm birth low-birth-weight infants complicated with pulmonary hypertension (Zheng et al., 2023; Sun et al., 2022; Chang et al., 2024). The underlying mechanism may be related to the improvement of pulmonary blood flow perfusion and the alleviation of inflammatory injury. Large-sample multicenter studies have found that routine iNO inhalation cannot reduce the incidence of BPD in high-risk preterm infants without pulmonary hypertension, on the contrary, it may increase the risk of oxidative stress due to the activation of inducible nitric oxide synthase (iNOS). Controversies surrounding the use of iNO for the treatment of BPD in these randomized controlled trials mainly arise from the complexity and heterogeneity of patients with BPD, as well as variations in disease phenotypes. Precise and individualized administration of iNO is expected to produce clinical benefits for vulnerable infants. In contrast, universal, indiscriminate iNO administration fails to improve infant outcomes and leads to unnecessary drug exposure and wasted medical resources. NO relaxes vascular smooth muscle cells, dilates pulmonary blood vessels, and optimizes the pulmonary ventilation-perfusion matching ratio. For infants with BPD driven primarily by pulmonary vascular lesions, iNO has a well-defined biological mechanism of action, and carefully selected BPD patients can achieve better prognoses following iNO therapy. Pulmonary vascular resistance in preterm infants follows a U-shaped curve across gestational ages: resistance is elevated in extremely preterm infants and near-term infants, while the lowest resistance is seen in infants born at 30–31 weeks of gestation. This pattern matches the clinical utilization trend of iNO: iNO is far more frequently administered to extremely preterm infants with a gestational age below 25 weeks, whereas its use is less common in preterm infants of middle gestational age. iNO exerts limited therapeutic effects in two groups of patients: preterm infants with poorly developed pulmonary vascular smooth muscle, immature small-caliber pulmonary vasculature, blunted vascular responsiveness and inherently high pulmonary vascular resistance; and infants with BPD complicated by insufficient cGMP activity, oxidative stress, or abnormal blood flow distribution. Current studies investigating the initial dose of iNO for preterm infants show substantial heterogeneity, and no consistent, effective consensus has been reached. The standard initial iNO dose widely used in preterm infants is 5 ppm, with continuous monitoring of fraction of inspired oxygen and arterial oxygen saturation. If no clinical improvement is observed, the dose can be titrated up to 10 ppm at 5–10 min intervals, with a maximum dose of 20 ppm. If there is no therapeutic response at 20 ppm, or if the infant develops worsening oxygenation, blood pressure, heart rate or peripheral perfusion, the iNO dose should be rapidly tapered and discontinued within minutes. Most infants demonstrate improvements in clinical manifestations and echocardiographic parameters at 5 ppm. Low-dose iNO prevents drastic fluctuations in physiological indicators and reduces the risk of complications including intraventricular hemorrhage. Current available evidence does not support routine early use of iNO for the purpose of preventing BPD (Bischoff, 2026). Inhalation of high-concentration iNO (>20 ppm) may lead to adverse reactions such as methemoglobinemia and aggravated lung injury (Takahashi and Murase, 2024). Therefore, while focusing on the therapeutic effects of iNO, its potential adverse effects should not be ignored (Feng et al., 2024). After entering the blood, NO binds irreversibly to ferrous ions in hemoglobin to form methemoglobin. Preterm infants possess an immature antioxidant metabolic enzyme system, with a clearance capacity only one-third that of term infants (Cookson and Kinsella, 2024). Infants with BPD frequently experience persistent hyperoxia exposure, which synergistically exacerbates the *in vivo* accumulation of methemoglobin, impairs the oxygen-carrying capacity of hemoglobin, and consequently triggers tissue hypoxia. Randomized controlled clinical trials have demonstrated an elevated risk of methemoglobinemia in neonates receiving high-concentration inhaled NO therapy, whereas the incidence decreases markedly with conventional low-dose regimens (Feng et al., 2024; Group, 1997). Accordingly, routine monitoring of methemoglobin is recommended for all infants administered inhaled nitric oxide. Inhaled NO directly or indirectly induces oxidative stress and cytotoxicity in pulmonary epithelial cells. Coexistence of nitric oxide with high-concentration oxygen enables spontaneous oxidation to produce nitrogen dioxide, and higher concentrations of iNO correspond to greater nitrogen dioxide formation. As a potent inflammatory irritant, nitrogen dioxide disrupts alveolar epithelial cells, impairs pulmonary surfactant, and exacerbates pulmonary interstitial edema and fibrosis. Long-term high-dose nitric oxide administration induces pulmonary vascular tolerance to exogenous NO and suppresses endogenous NO synthesis. Abrupt discontinuation of treatment may lead to an abrupt elevation in pulmonary artery pressure, and even shock, resulting in hypoxia rebound crisis (Gien et al., 2020).

### 4.2 Molecular mechanisms of carbon monoxide (CO) in BPD-related lung injury

Low-dose inhaled CO is not incorporated into routine or standardized clinical protocols for the management of BPD in neonates. Its research application remains limited to *in vitro* experiments and animal models to date. Studies in animal models have demonstrated that CO ameliorates BPD-related lung injury. Its protective effects are mediated through two primary potential mechanisms. First, CO alleviates BPD-related lung injury by suppressing inflammatory responses. Second, it exerts pulmonary protective effects via inhibiting apoptosis of alveolar epithelial and pulmonary vascular endothelial cells.

#### 4.2.1 Inhibiting inflammatory responses to alleviate lung tissue injury

The occurrence of BPD is closely associated with cascading oxidative stress and excessive inflammatory responses triggered by stimuli such as hyperoxia exposure and mechanical ventilation in the lung tissues of preterm infants. These responses lead to damage of alveolar epithelial cells and pulmonary vascular endothelial cells, thereby impeding alveolar development. CO exerts a lung-protective effect by activating the intracellular antioxidant system, and the nuclear factor erythroid 2-related factor 2 (Nrf2)/heme oxygenase-1 (HO-1) axis serves as a critical pathway mediating CO’s protective role against BPD-related lung injury (Amata et al., 2017; Zhao et al., 2023). CO can activate Nrf2 through positive feedback regulation, promoting the translocation of Nrf2 from the cytoplasm to the nucleus. In the nucleus, Nrf2 binds to antioxidant response elements (AREs), upregulating the expression of heme oxygenase-1 (HO-1) and other antioxidant genes. This forms a “CO-HO-1″ protective loop, which significantly enhances the capacity of lung tissues to scavenge reactive oxygen species (ROS) and alleviates hyperoxia-induced oxidative stress injury.

In animal models of BPD, inhaled carbon monoxide suppresses inflammatory responses and ameliorates alveolar simplification (Anyanwu et al., 2014; Amata et al., 2017; Yang et al., 2023; Li et al., 2024; Zhuang et al., 2010). In hyperoxia-induced neonatal mouse models of BPD, the expression of HO-1 is significantly upregulated (Amata et al., 2017; Yang et al., 2023; Li et al., 2024). Exogenous CO therapy reduces hyperoxia-induced alveolar simplification, inhibits neutrophil and macrophage infiltration, and attenuates pulmonary inflammation. Studies on hyperoxia-induced mouse models have demonstrated that inhalation of low-concentration CO (250 ppm) downregulates the hyperoxia-induced overexpression of pro-inflammatory cytokines (including TNF-α, IL-1β, IL-6, CCL-2, CXCL1, and CXCL2) while upregulating the expression of anti-inflammatory cytokines IL-10 and IL-13, thereby significantly alleviating alveolar simplification in mice (Anyanwu et al., 2014). These findings indicate that HO-1 exerts a protective effect in hyperoxia-induced BPD mice, similar to that of exogenous CO in hyperoxia-exposed mice. HO-1 knockout mice exhibit BPD-like lung structural defects including alveolar simplification, pulmonary vascular dysplasia and increased macrophage infiltration even under normoxia (Anyanwu et al., 2014; Zhuang et al., 2010). Overexpression of HO-1 increases pulmonary vascular density in hyperoxia-exposed mice, mitigating pulmonary vascular remodeling and right ventricular hypertrophy (Fernandez-Gonzalez et al., 2012). This indirectly supports the protective role of the CO-HO-1 pathway against BPD-related lung damage.

Other *in vivo* and *in vitro* lung injury studies verify that carbon monoxide suppresses inflammation and mitigates lung injury, indirectly supporting its pulmonary protective properties. Research on hyperoxia-induced lung injury models has identified that the mitogen-activated protein kinase 3 (MKK3)/mitogen-activated protein kinase (MAPK) pathway mediates the protective effect of CO against hyperoxia-induced lung injury (Otterbein et al., 2003). Hyperoxia activates the MAPK pathway, leading to increased protein expression of extracellular signal-regulated kinase 1/2 (ERK1/ERK2), c-Jun N-terminal kinase (JNK), MKK3/6, and p38 MAPK in lung tissues (Brouard et al., 2000; Zhan et al., 2003; Brouard et al., 2002). Compared with wild-type mice, MKK3 knockout (mkk3^−/−^) mice develop lung injury earlier after hyperoxia exposure, and CO fails to inhibit the expression of pro-inflammatory cytokines or exert its protective effect in these mice. In mechanical ventilation-induced lung injury models, CO has been shown to significantly reduce ventilation-induced elevation of TNF-α levels, neutrophil infiltration, and IL-1β release (Dolinay et al., 2004). Inhalation of CO decreases the levels of IL-1β (Anyanwu et al., 2014), monocyte chemoattractant protein-1 (MCP-1), and macrophage inflammatory proteins (Anyanwu et al., 2014) in lung homogenates, attenuates the ventilation-induced upregulation of early growth response gene-1 (Egr-1), and thus prevents ventilation-induced lung injury. In cultured lung epithelial cells (A549 cells), CO induces the activation of p38 MAPK and MKK3, enhancing cell viability, these effects can be reversed by p38 MAPK inhibitors (Ning et al., 2005; Des et al., 2005). *In vitro* studies have revealed that CO induces the expression of peroxisome proliferator-activated receptor γ (PPAR-γ) in macrophages (Kang et al., 2021; Haschemi et al., 2011). PPAR-γ is recognized as an anti-inflammatory mediator that antagonizes the effects of Egr-1 (Hoetzel et al., 2008). Therefore, CO exerts its lung-protective effect by upregulating PPAR-γ and downregulating Egr-1 expression.

#### 4.2.2 Anti-apoptotic effects to protect alveolar epithelial and pulmonary vascular endothelial cells

During the development and progression of BPD, increased apoptosis of alveolar epithelial cells (AECs) and pulmonary vascular endothelial cells (PVECs) leads to alveolar structural damage and vascular bed reduction, resulting in alveolar developmental arrest. CO inhibits cell apoptosis through multiple pathways, maintaining cellular homeostasis in lung tissue. The mitochondrial pathway is a core regulatory site for cell apoptosis. Oxidative stress and inflammation can induce a decrease in mitochondrial membrane potential, promote the translocation of pro-apoptotic proteins Bax and Bak to the mitochondrial membrane, trigger the release of cytochrome C, and activate the caspase-9/caspase-3 apoptotic pathway. CO stabilizes mitochondrial membrane potential, inhibits the mitochondrial translocation of Bax, and upregulates the expression of anti-apoptotic proteins Bcl-2 and Bcl-xL, thereby blocking the activation of the mitochondrial apoptotic pathway (Ryter et al., 2018; Faller and Hoetzel, 2012). *In vitro* experiments have confirmed that the apoptosis rate of human type II alveolar epithelial cells (A549) cultured under hyperoxic conditions is significantly increased, whereas treatment with CO donors reduces caspase-3 activity, decreases cell apoptosis rate, and improves cell survival rate. Moreover, CO downregulates the expression of death receptors (e.g., TNF receptor 1, Fas), reduces ligand-receptor binding, and inhibits caspase-8 activation, blocking extrinsic apoptotic signal transduction (Zhang et al., 2003).

#### 4.2.3 Limitations of inhaled CO therapy for BPD in preterm infants

Low-dose CO exerts anti-inflammatory effects, promotes alveolarization, and ameliorates pulmonary vascular remodeling in animal models of BPD and *in vitro* cellular models. Nevertheless, its clinical application in preterm infants is subject to multiple non-negligible limitations. First, CO binds to hemoglobin with high affinity, which easily induces occult tissue hypoxia. The immature brain, retina and myocardium are at risk of long-term irreversible injury, and long-term follow-up data on neurodevelopmental safety in preterm infants remain absent. Second, the therapeutic window of CO is extremely narrow (the safe concentration range is limited to 25–100 ppm with intermittent inhalation). There are no standardized neonatal delivery devices, unified dosage regimens or individualized titration criteria for CO administration. Repeated arterial blood sampling for carboxyhemoglobin (COHb) monitoring brings additional trauma to preterm infants. Third, evidence from clinical trials is insufficient, with no large-scale multicenter randomized controlled trials to support its use. Current domestic and international clinical guidelines for neonatal BPD do not recommend inhaled CO therapy. Fourth, CO is a highly toxic hazardous gas. Neonatal intensive care units (NICUs) lack supporting facilities including negative pressure systems, waste gas recovery equipment and ambient gas monitoring devices, posing a poisoning risk to medical staff and other infants housed in the same unit. Fifth, there is a translational efficacy gap between simple hyperoxia-induced animal models and clinically complex multifactorial BPD. In addition, inhaled CO cannot achieve lung-targeted intervention; systemic vasodilation triggered by CO may aggravate preterm-specific complications such as hypotension, patent ductus arteriosus (PDA) and intracranial hemorrhage. In conclusion, inhaled carbon monoxide is currently only an investigational research tool and is not yet ready for routine clinical practice (Choi et al., 2022; Yuan et al., 2022).

### 4.3 Molecular mechanisms of hydrogen sulfide (H_2_S) in BPD-related lung injury

The gas signaling molecule H_2_S participates in alveolarization in animal models of BPD, alleviates pulmonary hypertension, suppresses inflammatory responses, and facilitates epithelial repair. Studies using hyperoxia-induced mouse model of BPD have demonstrated that exogenous H_2_S donors (e.g., GYY4137) markedly ameliorate impaired alveolarization, increase alveolar counts and reduce alveolar cavity dilation in neonatal mouse, while concurrently attenuating pulmonary hypertension and right ventricular hypertrophy (Madurga et al., 2015). *In vivo* experiments confirm that cystathionine β-synthase (CBS) and cystathionine γ-lyase (Cth) are the key enzymes responsible for endogenous H_2_S biosynthesis, which are expressed in respiratory epithelial cells and pulmonary vasculature. Moreover, during alveolarization in mice, CBS and Cth coordinately regulate angiogenesis essential for normal alveolar development. Both CBS-knockout and Cth-knockout mouse models exhibit reduced total alveolar number, enhanced muscularization of small pulmonary vessels and thickened vascular walls, validating the critical role of H_2_S in lung development. Studies investigating mechanical ventilation-induced lung injury have verified that H_2_S significantly reduces alveolar wall thickness and inflammatory cell infiltration, downregulates pulmonary levels of nuclear factor-α (NF-α), interleukin-1α/β (IL-1α/β), interleukin-2 (IL-2) and macrophage inflammatory protein-1 (MIP-1) (Wang et al., 2017; Veskemaa et al., 2025; Fa et al., 2010), inhibits nuclear factor-κB (NF-κB) activation, and suppresses reactive oxygen species (ROS) production in lung tissue (Ge et al., 2019). Notably, the Akt signaling pathway mediates the protective effects of H_2_S against ventilation-induced lung injury (Spassov et al., 2017). In a murine acute lung injury model induced by high-tidal-volume ventilation, H_2_S prevents ventilator-associated lung injury via activating Nrf2-dependent transcription of antioxidant genes (Francis et al., 2011). Findings from the above animal models indirectly elucidate the mechanistic basis underlying H_2_S-mediated protection against BPD-related lung injury. *In vitro* cellular studies reveal that H_2_S donors inhibit alveolar leukocyte infiltration and restore physiological levels of interleukin-10 (IL-10). Additionally, H_2_S accelerates the migration of type II alveolar epithelial cells (ATII cells), an effect abrogated by glibenclamide. These observations indicate that H_2_S exerts protective effects by boosting the production of anti-inflammatory cytokines, restricting the accumulation of macrophages and neutrophils in hyperoxia-induced alveolar airspaces, promoting ATII cell migration and wound repair through glibenclamide-sensitive ion channels, and potentially activating the lung-protective Akt signaling cascade (Madurga et al., 2014). In an *in vitro* model of hyperoxia-induced injury in human pulmonary artery endothelial cells, treatment with GYY4137 robustly augments capillary-like network formation and diminishes intracellular reactive oxygen species (ROS) generation (Vadivel et al., 2014). Collectively, accumulating evidence from these studies suggests that H_2_S confers protective effects against BPD-related lung injury by inhibiting pulmonary inflammation, cellular apoptosis and oxidative stress.

Numerous studies have demonstrated elevated ROS levels in preterm infants with BPD, and animal models of BPD have further identified ROS as a central mediator of lung injury. N-acetylcysteine (NAC), one of the most extensively investigated agents among existing therapeutic candidates, acts as a glutathione precursor and thiol donor. Double-blind controlled clinical trials in preterm infants have validated that intravenous NAC infusion fails to reduce the incidence of BPD or ameliorate pulmonary function (Ahola et al., 2003). Similar to carbon monoxide, H_2_S lacks randomized controlled clinical trials in neonates to verify its clinical benefits to date. H_2_S possesses a narrow physiological safety window ranging from 10 to 100 μmol/L. Preterm infants exhibit markedly higher susceptibility to H_2_S toxicity compared with adult animals, such that safe dosages established in animal models cannot be directly extrapolated to preterm neonates. Furthermore, gaseous H_2_S poses hazards during storage and transportation, with difficulties in precise dose titration and potential local irritant adverse reactions upon administration. H_2_S donors including sodium hydrosulfide and GYY4137 lack available data on pharmacokinetics and biodistribution across visceral organs. Current real-time *in vivo* monitoring techniques for endogenous H_2_S remain underdeveloped and are incapable of detecting its bioactive concentrations within alveolar tissue and systemic circulation. Preterm infants complicated with BPD frequently receive multiple concurrent medications, conferring substantial risks of drug-drug interactions. Collectively, all the aforementioned obstacles hinder the clinical translational application of H_2_S.

### 4.4 Effects of the cross-talk among gas signaling molecules on BPD

The protective mechanisms of the three major gas signaling molecules in BPD-related lung injury do not exist in isolation. Instead, these molecules form a complex interactive network through synergy, antagonism, or upstream-downstream regulation. Centering on anti-inflammation, antioxidation, pro-angiogenesis and alveolarization, this network modulates the progression of BPD, its imbalance will exacerbate lung injury, and targeted combined intervention holds potential therapeutic value. NO, CO, and H_2_S as the most extensively studied gas signaling molecules in BPD, have been confirmed to exert protective effects, and their interaction is dominated by synergy and supplemented by antagonism, functioning through shared pathways or cross-regulation.

#### 4.4.1 NO and CO

The crosstalk between NO and CO is dose-dependent, exhibiting synergistic effects at low concentrations and antagonistic effects at high concentrations via distinct underlying mechanisms. At low concentrations, NO activates the Nrf2 signaling pathway to upregulate HO-1 transcription, thereby boosting endogenous CO production. In turn, CO stabilizes eNOS protein and alleviates eNOS uncoupling, which reduces toxic peroxynitrite formation and elevates the bioavailability of physiological NO. This creates a positive NO–HO-1–CO feedback loop that jointly eliminates hyperoxia-induced ROS and inhibits alveolar epithelial apoptosis. Both gas molecules exert vasodilatory effects on the pulmonary circulation, synergistically suppress pulmonary artery smooth muscle hypertrophy, and ameliorate BPD complicated with pulmonary hypertension (Choi and Kim, 2021). Under persistent excessive hyperoxic stimulation, overproduced NO and CO compete for binding sites on heme proteins. NO occupies heme-binding pockets and blocks downstream CO signaling; excess CO reciprocally suppresses NO-mediated cGMP synthesis, ultimately blunting their pulmonary vasodilatory capacity (Kim et al., 2023).

#### 4.4.2 NO and H_2_S

H_2_S acts as a sensitizer for NO signaling. H_2_S upregulates NO synthesis and restores sGC sensitivity. In preterm hyperoxic lung injury, sGC undergoes oxidative modification and subsequent inactivation, rendering NO biologically ineffective. H_2_S repairs the heme domain of sGC via thiol reductive modification, thereby restoring the NO–cGMP signaling cascade. Meanwhile, H_2_S activates the PI3K/Akt pathway to enhance eNOS activity, augment the production of physiological NO, suppress the overexpression of iNOS, and mitigate the generation of toxic oxidative NO metabolites (Lin et al., 2020). Animal studies have demonstrated that combined administration of the H_2_S donor GYY4137 and low-dose NO significantly elevates alveolar counts, attenuates airway smooth muscle hyperplasia, and reverses airway remodeling that fails to respond to monotherapy in neonatal mice. Both molecules inhibit NF-κB activation and reduce the release of IL-1β and TNF-α. H_2_S eliminates NO-derived peroxynitrite anions and interrupts the amplification of oxidative cascades, exerting synergistic protective effects on type II alveolar epithelial cells and preserving pulmonary surfactant secretion. These findings indicate that H_2_S and NO share synergistic antioxidant and anti-inflammatory properties (Ganguly et al., 2021; Bartman et al., 2024; Gheibi et al., 2018; Kolluru et al., 2016). However, high concentrations of H_2_S can react with NO to form thio-nitro derivatives, reducing the level of free NO and inhibiting vasodilation, resulting in an antagonistic effect (Davis and Pluth, 2025; Lee et al., 2018).

#### 4.4.3 CO and H_2_S

CO activates Nrf2 through HO-1, and H_2_S further stabilizes the nuclear translocation of Nrf2. This dual upregulation of antioxidant proteins (SOD, GSH) counteracts high-oxygen-induced lung injury. Both can jointly promote the proliferation of pulmonary microvascular endothelium, improve alveolarization, and reduce the typical “simplified alveolar structure' pathological changes of BPD. HO-1 is a necessary mediator for the protective effect of H_2_S. In neonatal mice with HO-1 knockout, H_2_S is unable to alleviate high-oxygen-induced lung injury, proving that the protective effect of H_2_S is partially dependent on endogenous CO production. The two can interactively regulate enzyme expression. H_2_S upregulates HO-1 expression and increases CO synthesis. CO slightly upregulates CSE enzyme, elevating the basal concentration of H_2_S in the lungs, forming a weak positive feedback loop. However, this loop is disrupted by NF-κB in severe inflammatory lungs, and the endogenous synthesis of both gases synchronously decreases (Amata et al., 2017; Zhao et al., 2023; Citi et al., 2026; Ali et al., 2022; Qingyou et al., 2004; Oh et al., 2006).

#### 4.4.4 NO, CO and H_2_S interacting network

HO-1/Nrf2 constitutes the core signaling pathway among the three. Both NO and H_2_S can independently induce HO-1 expression and promote CO production. CO, in turn, repairs the stability of eNOS and CSE enzymes, maintaining the endogenous synthesis of NO and H_2_S. The three jointly activate Nrf2 to form a triple antioxidant barrier, blocking the high-oxygen - ROS - inflammation cascade reaction, and is the core synergistic effect in inhibiting BPD occurrence. Under the pathological state of BPD, this axis is damaged, Nrf2 degradation accelerates, HO-1, eNOS, and CSE are simultaneously downregulated, and the endogenous generation of the three protective gas molecules is completely insufficient, forming a vicious cycle. H_2_S has the function of repairing sGC and increasing the vasodilatory effect of NO. CO has the effect of stabilizing the endothelial barrier and reducing pulmonary edema. The combination of the three inhibits pulmonary artery myogenesis, improves pulmonary microcirculation perfusion, and resolves the pulmonary vascular development blockage of BPD. NO reduces epithelial apoptosis, H_2_S promotes alveolar separation, and CO inhibits interstitial fibrosis. The three jointly maintain the survival of alveolar type II cells and restore the normal alveolar structure. The three have competitive interactions with mitochondrial cytochrome c oxidase. Low concentrations combined can stabilize the mitochondrial membrane potential and reduce mitochondrial ROS. However, if all three gases accumulate excessively simultaneously, it will competitively inhibit the respiratory chain, reduce ATP generation, and aggravate the energy damage of the immature lung. The regulation of the three pathways by inflammation is also bimodal. The three can jointly block the NF-κB and MAPK inflammatory pathways, reduce the release of pro-inflammatory factors, but in severe inflammation, iNOS is overactivated and produces a large amount of toxic NO, counteracting the anti-inflammatory and antioxidant effects of CO and H_2_S, and aggravating lung injury (Lin and Wang, 2020; Ali et al., 2022; Huwiler et al., 2023; Giuffrè and Vicente, 2018; Li et al., 2025; Liu et al., 2012).

## 5 Limitations and future directions

The pathogenesis of BPD-related lung injury is complex, with oxidative stress, inflammatory response, and abnormal development of blood vessels and alveoli as the core pathological processes. The three major gas signaling molecules—NO, CO, and H_2_S—exert significant lung-protective effects in alleviating oxidative stress, inhibiting inflammatory response, and promoting alveolar and vascular development by regulating key signaling pathways such as the Nrf2/HO-1 axis and NF-κB pathway. Current studies have confirmed that the three endogenous gas signaling molecules can alleviate BPD-related lung injury by regulating oxidative stress, inflammatory responses, and lung vascular and alveolar development. However, there are still various constraints and clinical translation difficulties in this field. The research conclusions regarding their regulatory mechanisms mostly come from BPD animal models and indirect *in vitro* experimental evidence. The BPD population of preterm infants shows heterogeneity in terms of gestational age, maternal factors, pathological classification, postnatal oxygen use, and treatment. Animal models cannot fully replicate human BPD. Gas molecules generally have a bidirectional effect of low concentration protection and high concentration toxicity. The complete dose-effect relationship has not been systematically clarified, and there is a lack of a unified *in vivo* monitoring method. The safe administration strategy has not yet been resolved. These all limit the clinical research of gas signaling molecules in BPD-related lung injury. Future research should focus more on refined and individualized analysis, relying on human lung organoids and standardized large animal models, combined with single-cell spatial multi-omics to analyze the cross-regulation pathways of gas signaling, establish unified detection standards, and develop targeted pulmonary sustained-release drug delivery systems. Conduct stratified cohort clinical trials to explore the synergistic schemes of multiple gases or combined antioxidants, and promote prenatal intervention and long-term follow-up, to promote the gas signaling molecule targets towards precise prevention and treatment of BPD. These efforts will facilitate the translation of basic research findings into clinical practice and ultimately improve the prognosis of preterm infants with BPD.
